# Impact of BMI and waist circumference on epigenome-wide DNA methylation and identification of epigenetic biomarkers in blood: an EWAS in multi-ethnic Asian individuals

**DOI:** 10.1186/s13148-021-01162-x

**Published:** 2021-10-20

**Authors:** Yuqing Chen, Irfahan Kassam, Suk Hiang Lau, Jaspal S. Kooner, Rory Wilson, Annette Peters, Juliane Winkelmann, John C. Chambers, Vincent T. Chow, Chiea Chuen Khor, Rob M. van Dam, Yik-Ying Teo, Marie Loh, Xueling Sim

**Affiliations:** 1 Saw Swee Hock School of Public Health, National University of Singapore and National University Health System, 12 Science Drive 2, #10-01, Tahir Foundation Building, Singapore, 117549 Singapore; 2 Life Sciences Institute, National University of Singapore, Singapore, Singapore; 3 Department of Microbiology and Immunology, Yong Loo Lin School of Medicine, National University of Singapore, Singapore, Singapore; 4 Department of Cardiology, Ealing Hospital, London North West Healthcare NHS Trust, Middlesex, UK; 5 Imperial College Healthcare NHS Trust, Imperial College London, London, UK; 6 MRC-PHE Centre for Environment and Health, Imperial College London, London, UK; 7 National Heart and Lung Institute, Imperial College London, London, UK; 8 Research Unit Molecular Epidemiology, Institute of Epidemiology, Helmholtz Zentrum München, German Research Center for Environmental Health, 85764 Neuherberg, Bavaria, Germany; 9 Institute of Epidemiology, Helmholtz Zentrum München, German Research Center for Environmental Health, Neuherberg, Germany; 10 German Center for Cardiovascular Research, Partner Site Munich Heart Alliance, Munich, Germany; 11 Institute of Neurogenomics, Helmholtz Zentrum München, Munich, Germany; 12 Institute of Human Genetics, Technical University of Munich, Klinikum rechts der Isar, Munich, Germany; 13 Lehrstuhl Für Neurogenetik, Technische Universität München, Munich, Germany; 14 Munich Cluster for Systems Neurology, Munich, Germany; 15 Lee Kong Chian School of Medicine, Nanyang Technological University, 11 Mandalay Road, Level 18, Lee Kong Chian Clinical Science Building, Singapore, 308232 Singapore; 16 Department of Epidemiology and Biostatistics, Imperial College London, London, UK; 17 National University Health System Infectious Diseases Translational Research Program, Department of Microbiology and Immunology, Yong Loo Lin School of Medicine, National University of Singapore, Singapore, Singapore; 18 Genome Institute of Singapore, Agency for Science, Technology and Research, Singapore, Singapore; 19 Singapore Eye Research Institute, Singapore National Eye Centre, Singapore, Singapore; 20 Department of Nutrition and Department of Epidemiology, Harvard T.H. Chan School of Public Health, Boston, MA USA; 21 National Skin Centre, Singapore, Singapore

**Keywords:** DNA methylation, Epigenome-wide association study, Obesity, Body mass index, Waist circumference, Inflammation, Metabolites

## Abstract

**Background:**

The prevalence of obesity and its related chronic diseases have been increasing especially in Asian countries. Obesity-related genetic variants have been identified, but these explain little of the variation in BMI. Recent studies reported associations between DNA methylation and obesity, mostly in non-Asian populations.

**Methods:**

We performed an epigenome-wide association study (EWAS) on general adiposity (body mass index, BMI) and abdominal adiposity (waist circumference, WC) in 409 multi-ethnic Asian individuals and replicated BMI and waist-associated DNA methylation CpGs identified in other populations. The cross-lagged panel model and Mendelian randomization were used to assess the temporal relationship between methylation and BMI. The temporal relationship between the identified CpGs and inflammation and metabolic markers was also examined.

**Results:**

EWAS identified 116 DNA methylation CpGs independently associated with BMI and eight independently associated with WC at false discovery rate *P*_FDR_ < 0.05 in 409 Asian samples. We replicated 110 BMI-associated CpGs previously reported in Europeans and identified six novel BMI-associated CpGs and two novel WC-associated CpGs. We observed high consistency in association direction of effect compared to studies in other populations. Causal relationship analyses indicated that BMI was more likely to be the cause of DNA methylation alteration, rather than the consequence. The causal analyses using BMI-associated methylation risk score also suggested that higher levels of the inflammation marker IL-6 were likely the consequence of methylation change.

**Conclusion:**

Our study provides evidence of an association between obesity and DNA methylation in multi-ethnic Asians and suggests that obesity can drive methylation change. The results also suggested possible causal influence that obesity-related methylation changes might have on inflammation and lipoprotein levels.

**Supplementary Information:**

The online version contains supplementary material available at 10.1186/s13148-021-01162-x.

## Introduction

In the past two decades, the prevalence of obesity has been on the rise in Asian countries in parallel with rapid economic growth [1, 2]. Obesity is a well-established risk factor for many common diseases such as diabetes [3, 4], hypertension [5], and cardiovascular diseases [6]. Body mass index (BMI) is a common and easily measured indicator for general obesity. Compared to Europeans, Asians have lower average BMI but more total and visceral fat and are more likely to develop type 2 diabetes (T2D) at a lower BMI [7]. Waist circumference (WC) as a measure of abdominal obesity has also been associated with a higher T2D risk [8–10]. Both general and abdominal obesity are influenced by genetic and lifestyle factors such as intakes of energy-dense food and lack of physical activity [11–14].

Genome-wide association studies (GWAS) have identified over 900 genetic loci associated with BMI [15–17], which explained only ~ 6% of the variation and ~ 25% of the SNP-heritability of BMI. This has led to evolving interest in epigenetics to explain some of the missing heritability. Epigenetic mechanisms include DNA methylation, chromosome histone modification, and noncoding RNA regulation. DNA methylation is one of the most well-studied epigenetic mechanisms that blocks transcription factors from binding to promoters by adding a methyl group to the carbon C5 of cytosine nucleotides and subsequently alter gene expression [18]. Using commercial arrays to profile epigenome-wide DNA methylation, more than 5,000 DNA methylation sites have been identified to be associated with obesity-related traits [19]. For example, cg00574958 located in *CPT1A* on chromosome 11 was consistently hypomethylated with increased BMI in multiple studies [20–22].

Due to the plasticity of DNA methylation in response to environmental changes, it is possible that DNA methylation changes can be a consequence of BMI [23]. The temporal relationship between BMI and DNA methylation has been assessed using Mendelian randomization and structural equation modeling in European, South Asian, and African-American populations [22, 24, 25]. While most of the evidence supports the hypothesis that methylation changes are a consequence of obesity, there is also evidence of CpGs having a causal effect on BMI changes [22, 24]. In addition, gene set analyses on genes nearest to the identified BMI-associated methylation markers showed enrichment in a diverse range of biological processes including lipid metabolism, amino acids transport, neuronal function, and inflammatory pathways [22, 24, 26]. For example, DNA methylation at *CETP* and *LPL* was associated with gene expression and lipoproteins levels in people with obesity [27]. In addition, hypermethylation at *IL-6* was found in Korean women with obesity [28]. Consistent with this finding, the activity of DNA methyltransferase isoforms and global DNA hypomethylation were decreased in interleukin 6 (IL-6)-induced insulin resistant human endothelial cells [29]. These observations indicate that DNA methylation can influence biological pathways involved in the development of obesity-related diseases.

Previous epigenome-wide association studies (EWAS) on obesity were conducted in populations of predominantly European ancestry, and it remains unclear if these findings may be transferable to Asian populations who have a different propensity to develop metabolic diseases for the same BMI. Therefore, to identify BMI-associated CpGs and to understand the link between BMI and DNA methylation in Asians, we conducted a cross-sectional analysis of the epigenome-wide associations of BMI and WC among 409 multi-ethnic Asian individuals (228 Chinese, 84 Malay, 97 Indian). We identified 116 BMI-associated CpGs (including six novel sites) and eight WC-associated CpGs (including two novel sites) that reached epigenome-wide significance (false discovery rate *P*_FDR_ < 0.05). Our results suggest that BMI is likely to be a cause rather than a consequence of DNA methylation change at the identified loci in a longitudinal setting in Chinese individuals. Finally, to assess the clinical relevance of methylation involved in the obesity-related inflammatory and metabolic alteration, we tested adiposity-associated methylation markers for association with inflammation markers IL-6 and metabolic biomarkers such as lipoproteins.

## Results

### Study samples characteristics

**Fig. 1:**
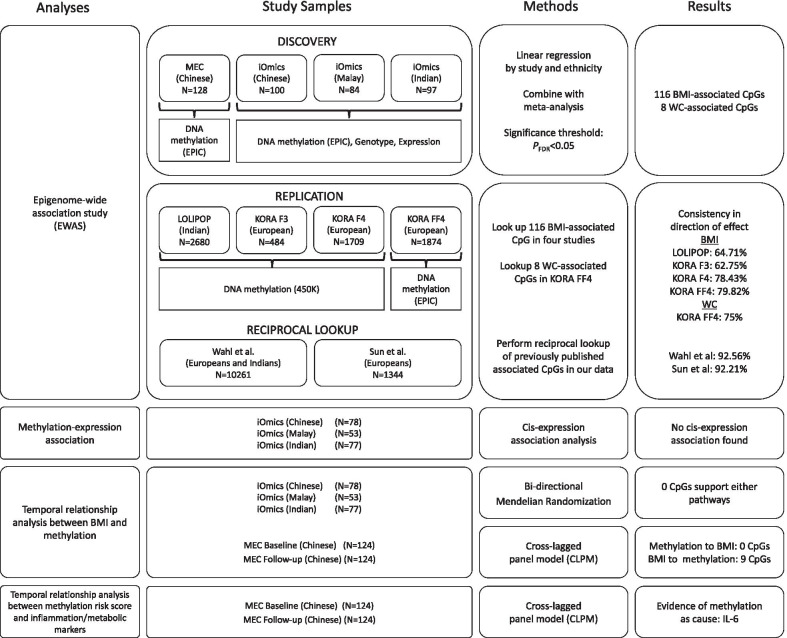
Summary of analyses, study samples, methods, and results in this study. MEC, Multi-Ethnic Cohort; iOmics, Singapore Integrative Omics Study; LOLIPOP, The London Life Sciences Prospective Population Study; KORA, Cooperative Health Research in the Region of Augsburg; 450 K, Illumina Infinium HumanMethylation450 BeadChip array; EPIC, Illumina Infinium HumanMethylation EPIC array; BMI, body mass index; WC, waist circumference; IL-6, interleukin 6

Summary of analyses, study samples, methods, and results in this study are shown in Fig. 1. All samples included in this study were selected from the Multi-Ethnic Cohort (MEC) [30], including 286 participants (102 Chinese, 86 Malay, 98 Indian) at their first follow-up from the Singapore Integrative Omics Study (iOmics) [31], and 140 Chinese individuals from MEC with both baseline and first follow-up measurements, hereafter referred to as ‘MEC Chinese sample’ set. Summary characteristics of 264 MEC Chinese and 281 iOmics participants after quality control (QC) are summarized in Additional file 1: Table S1. There was a significant difference in BMI across ethnic groups in the iOmics study (*P*_ANOVA_ = 1.31 × 10^–16^), whereas no difference was observed between the MEC Chinese and iOmics Chinese (*P*_*t*-test_ = 0.83).

### EWAS of adiposity and meta-analysis

**Fig. 2:**
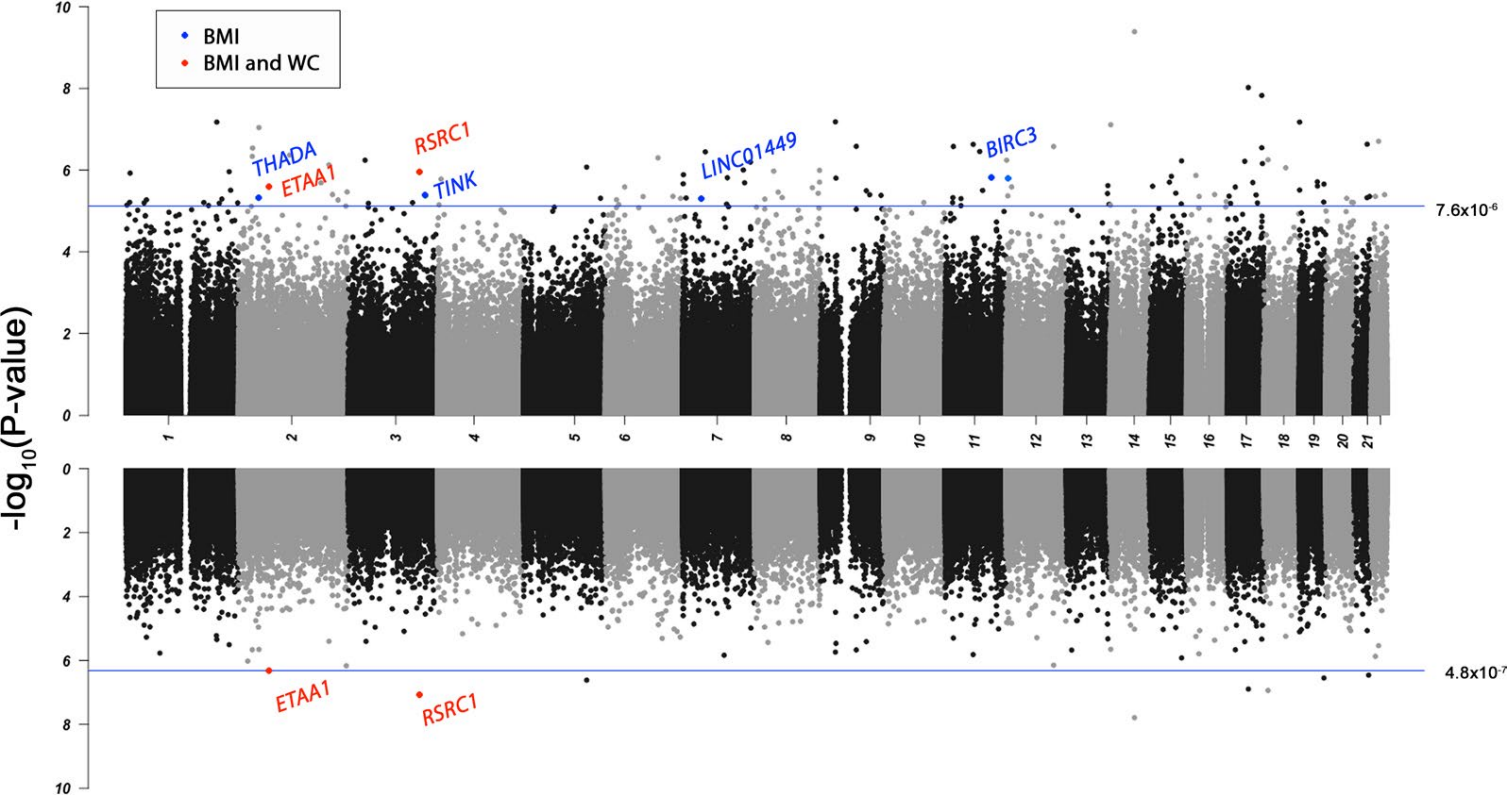
Genome-wide mirror Manhattan plot of association statistics from epigenome-wide association analysis in multi-ethnic Asians with BMI association results on the upper panel and WC association results on the lower panel. At an epigenome-wide significance of *P*_FDR_ < 0.05, we identified 116 BMI-associated and 8 WC-associated CpGs. Novel associations were labeled with the nearest candidate gene and colored by trait. Novel CpGs near *THADA*, *TNIK*, *LINCO1449*, and *BIRC3* were associated with BMI only, while CpGs near *ETAA1* and *RSRC1* were associated with both BMI and WC

To identify central (BMI) and abdominal (WC) adiposity-associated DNA methylation, we performed EWAS in 281 iOmics (100 Chinese, 84 Malays, and 97 Indians) and 128 MEC Chinese samples (Additional file 1: Table S2). We performed association analysis between BMI and CpGs in the two sets of samples separately by ethnicity and combined them with fixed-effect meta-analysis. We first meta-analyzed Chinese samples and identified 29 CpGs significantly associated with BMI (*P*_FDR_ < 0.05) (Additional file 1: Table S3). We then meta-analyzed the combined Chinese results with the iOmics Malay and Indian association results. A total of 123 CpGs at 116 loci were significantly associated with BMI (*P*_FDR_ < 0.05) after meta-analysis (Additional file 1: Table S3). Conditional analysis of seven loci containing two BMI-associated CpGs did not identify secondary signals. Of the 29 CpGs identified in Chinese meta-analysis, 21 remained significant in the trans-ethnic meta-analysis. We observed heterogeneity across ethnicity (*P*_het_ < 0.05) at 3 CpGs (cg17544521 in *CACNA1S*, cg06190406 in *OBSCN*, and cg20585768 in *LCLAT1)*, where the association was mainly driven by Chinese. Among the 116 independent BMI-associated CpGs, 110 CpGs have been previously associated with BMI in other populations as reported in two EWAS catalogues, Atlas and MRC-IEU. We identified six novel BMI-associated CpGs, namely cg15103625 near *THADA*, cg07421368 on the body of *ETAA1*, cg08010984 on the body of *TNIK*, cg15103625 within 1,500 bp from the transcript start site (TSS1500) of *RSRC1*, cg16309866 near *LINC01449*, and cg19120513 on the 5’ untranslated regions (5’UTR) of *BIRC3* (Fig. 2, Table 1). The strongest signal in our meta-analysis was cg10919522 in *C14orf43* (*P*_meta_ = 4.07 × 10^–10^)*,* which was also reported in EWAS of Europeans populations [24, 25]. Twenty-four CpGs were at/near obesity-related genes including three of the six novel BMI-associated CpGs, namely cg02871985 near *THADA*, cg08010984 in *TNIK*, and cg15103625 in *RSRC1*. cg08010984 in *TNIK* was inversely associated with BMI (*P*-value = 4.26 × 10^–6^) and the association was predominantly driven by Chinese (*β*_Chinese_ = − 27.24, *β*_Malay_ = − 19.62, *β*_Indian_ = − 18.96, *P*_Chinese_ = 2.14 × 10^–7^, *P*_Malay_ = 0.23, *P*_Indian_ = 0.16). cg15103625 in *RSRC1* was directly associated with BMI (*P*_meta_ = 1.12 × 10^–6^), and consistent across ethnicities (*P*_het_ = 0.70). We identified eight CpGs significantly associated with WC (Additional file 1: Table S3), of which two, cg15103625 in *RSRC1* and cg07421368 in *ETAA1*, were novel for both BMI and WC. Seven of the WC-associated CpGs overlapped with BMI-associated CpGs.

**Table 1:** Epigenome-wide association identified six novel BMI-associated CpGs and two WC-associated CpGs (*P*_FDR_ < 0.05) in Asian samples

CpG	Chr	Position	Nearest gene	Trait	Relation to Island	Location in gene	MEC Chinese				
							Beta Mean	Beta SD	Effect	SE	P
**cg02871985**	2	43,398,154	***THADA***	BMI	Island	Intergenic	0.11	0.02	− 21.61	22.03	3.29E−01
**cg07421368**	2	67,624,930	*ETAA1*	BMI	S_Shore	Body	0.02	0.01	41.17	60.71	4.99E−01
**cg07421368**	2	67,624,930	*ETAA1*	WC	S_Shore	Body	0.02	0.01	− 32.44	157.58	8.37E−01
cg15103625	3	157,826,388	***RSRC1***	BMI	N_Shore	TSS1500	0.16	0.03	27.79	13.01	3.48E−02
cg15103625	3	157,826,388	***RSRC1***	WC	N_Shore	TSS1500	0.16	0.03	75.44	33.63	2.69E−02
cg08010984	3	170,964,901	***TNIK***	BMI	OpenSea	Body	0.43	0.05	− 27.83	7.95	6.64E−04
cg16309866	7	41,264,719	*LINC01449*	BMI	OpenSea	Intergenic	0.53	0.05	− 33.93	9.94	8.94E−04
cg19120513	11	102,189,303	*BIRC3*	BMI	S_Shore	5'UTR	0.38	0.05	− 28.23	11.33	1.42E−02

CpG	iOmics Chinese					Chinese meta-analysis				iOmics Indian				
	Beta Mean	Beta SD	Effect	SE	P	Effect	SE	P	P_het_	Beta Mean	Beta SD	Effect	SE	P
**cg02871985**	0.18	0.02	− 57.56	21.15	7.91E−03	− 40.31	15.26	8.24E−03	2.39E−01	0.18	0.03	− 56.94	16.23	7.45E−04
**cg07421368**	0.05	0.04	43.10	8.66	3.43E−06	43.06	8.58	**5.16E−07**	9.75E−01	0.05	0.01	− 27.33	58.40	6.41E−01
**cg07421368**	0.05	0.04	114.79	21.47	7.56E−07	112.10	21.27	1.36E−07	3.55E−01	0.05	0.01	− 29.78	134.76	8.26E−01
cg15103625	0.28	0.04	40.45	10.43	2.09E−04	35.49	8.14	1.29E−05	4.48E−01	0.27	0.03	36.50	17.87	4.45E−02
cg15103625	0.28	0.04	102.90	26.25	1.80E−04	92.50	20.69	7.83E−06	5.20E−01	0.27	0.03	105.78	40.56	1.09E−02
cg08010984	0.54	0.05	− 26.15	10.84	1.81E−02	− 27.24	6.41	2.14E−05	9.01E−01	0.53	0.05	− 18.96	13.22	1.55E−01
cg16309866	0.60	0.05	− 47.72	13.29	5.57E−04	− 38.87	7.96	**1.05E−06**	4.06E−01	0.61	0.05	− 4.81	16.94	7.77E−01
cg19120513	0.47	0.05	− 35.98	14.83	1.74E−02	− 31.09	9.00	5.53E−04	6.78E−01	0.45	0.06	− 34.31	15.82	3.31E−02

CpG	iOmics Malay					Trans-ethnic meta-analysis				
	Beta Mean	Beta SD	Effect	SE	P	Effect	SE	P	P_het_	P_FDR_
**cg02871985**	0.18	0.02	− 46.00	31.00	1.43E−01	− 47.88	10.46	**4.75E−06**	5.83E−01	0.043
**cg07421368**	0.05	0.01	− 114.23	76.50	1.40E−01	39.68	8.43	**2.55E−06**	1.37E−01	0.035
**cg07421368**	0.05	0.01	− 148.44	176.17	4.03E−01	105.05	20.86	**4.77E−07**	2.57E−01	0.048
cg15103625	0.28	0.04	19.14	16.35	2.46E−01	32.85	6.75	**1.12E−06**	6.99E−01	0.025
cg15103625	0.28	0.04	60.89	36.86	1.03E−01	88.37	16.49	**8.32E−08**	7.54E−01	0.026
cg08010984	0.55	0.04	− 19.62	16.18	2.30E−01	− 24.98	5.43	**4.26E−06**	9.28E−01	0.043
cg16309866	0.60	0.05	− 18.86	27.56	4.96E−01	− 31.83	6.97	**4.99E−06**	2.37E−01	0.043
cg19120513	0.46	0.06	− 51.67	18.94	8.17E−03	− 34.76	7.23	**1.53E−06**	7.68E−01	0.029

CpGs on 450k array and obesity-related genes are highlighted in bold. cg02871985 is probe-in-SNP

Chr, Chromosome; S_Shore, South Shore; N_Shore, North Shore; Body, gene body; TSS1500, within 1500 bp from transcription start site; UTR, untranslated regions; FDR, false discovery rate; SE, standard error; Phet, *P*-value for heterogeneity test

To compare previously reported BMI-EWAS associations with our data, we first performed lookups of 254 BMI-associated CpGs from Wahl et al. [24] in Europeans/South Asians and 349 BMI-associated CpGs from Sun et al. [25] in Europeans/African Americans in our meta-analysis (Additional file 1: Table S4). In the first set of 254 BMI-associated CpGs, 242 CpGs passed QC in our data and 224 CpGs (92.56%) showed consistency in direction of effects (binomial test *P*-value < 2.2 × 10^–16^), and 13 CpGs were associated with BMI in our meta-analysis at Bonferroni-corrected *P*-value (0.0002 = 0.05/242). Similarly, in the second set of 349 BMI-associated CpGs, 321 CpGs passed QC, and 296 CpGs (92.21%) showed consistent direction of effects (binomial test *P*-value < 2.2 × 10^–16^) with ten CpGs associated with BMI in our meta-analysis at Bonferroni-corrected *P*-value (0.00016 = 0.05/321). Six CpGs overlapped and passed Bonferroni-corrected *P*-value in both datasets. When we looked up the 116 BMI-associated CpGs and eight WC-associated CpGs identified in our meta-analysis in The London Life Sciences Prospective Population Study (LOLIPOP), Cooperative Health Research in the Region of Augsburg from the first follow-up of the S3 survey (KORA F3), and the first and second follow-ups of the S4 survey (KORA F4, KORA FF4), 114 were presented in KORA FF4 while only 51 were present in LOLIPOP and KORA F3 and F4 due to difference in array coverage (Additional file 1: Table S5). Out of the overlapping CpGs, 33 CpGs (64.71%) in LOLIPOP South Asian, 32 CpGs (62.75%) in KORA F3, 40 CpGs (78.43%) in KORA F4, and 91 CpGs (79.82%) in KORA FF4 showed consistent direction of effect (binomial test *P*-value = 0.049, 0.09, 5.7 × 10^–6^ and 9.3 × 10^–11^, respectively). We also looked up eight WC-associated in KORA FF4 and found 6 CpGs (75%) with consistent direction of effect (binomial test *P*-value = 0.29).

### Association of DNA methylation and gene expression

To determine whether the identified BMI-associated CpGs may influence gene expression in blood, we examined the association between the identified 116 BMI-associated CpGs and 4,162 transcripts located within 1 Mb of the corresponding CpGs in a subset of iOmics samples with expression data available (total *n* = 208: 78 Chinese, 53 Malay, 77 Indian). No cis-transcripts were significantly associated with methylation level at *P*_FDR_ < 0.05. For the six novel BMI-associated CpGs identified, cg02871985 was positively nominally associated with the expression level of *THADA* (*P*-value = 0.046). cg08010984 annotated near *TNIK* and cg19120513 near *BIRC3* were negatively nominally associated with the expression level of *RNU6-348P* (*P*-value = 0.045) and *MMP8* (*P*-value = 0.006).

### Temporal relationship analysis of BMI and DNA methylation

#### Cross-lagged panel model

To further explore the link between BMI and DNA methylation, we examined the temporal association between BMI and the identified 116 CpGs using the cross-lagged panel model (CPLM) in 124 MEC Chinese samples with multiple timepoints. Among the 116 CpGs that showed significant association with BMI in meta-analysis, the path coefficients from baseline BMI to follow-up DNA methylation were nominally significant at 92 CpGs (79% of 116; *P*_FDR_ < 0.05). In contrast, no CpG showed significant path coefficient from baseline DNA methylation to follow-up BMI (Additional file 1: Table S6), suggesting that BMI has a causal effect on methylation change.

#### Bidirectional Mendelian randomization (MR)

Forward and backward MR were performed in a subset of 208 iOmic samples with genotype available (78 Chinese, 53 Malay, 77 Indian) by using genetic variants as instrumental variables (IV) to study the temporal relationship between methylation and BMI. In the forward MR, we identified 94 cis-SNPs associated with 116 BMI-associated CpGs, where the effect sizes on BMI were obtained from GWAS summary statistics in over 170,000 Japanese from Biobank Japan [16]. By calculating the predicted effect between CpG and BMI, we found no CpG indicative of causal effect of methylation on BMI (Bonferroni-adjusted *P*-value threshold of 5 × 10^–4^, min *P*-value = 1.1 × 10^–3^, Additional file 1: Table S7). In the backward MR, the reverse causality was investigated using polygenic risk score (PRS) as IV. The PRS was calculated using 85 previously reported BMI-associated SNPs in Biobank Japan (Additional file 1: Table S8). No CpG supported a causal link from BMI to methylation (min *P*-value = 0.027). The correlation between predicted and observed effects in forward MR was − 0.01 (*P*-value = 0.90) for methylation as cause, and 0.43 (*P*-value = 1.46 × 10^–6^) in backward MR for BMI as cause.

### Temporal relationship analysis of methylation with inflammation markers and metabolomics biomarkers

**Fig. 3:**
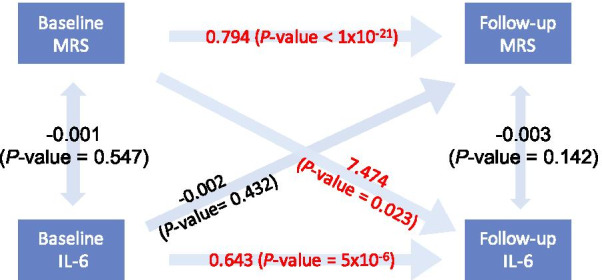
Cross-lagged panel analysis (CLPM) of BMI-associated methylation risk score (MRS) and IL-6. Pathway coefficients and corresponding *P*-values are provided. Unidirectional arrows indicate regression, and bidirectional arrows indicate correlation. Arrows with red numbers indicate significant pathway coefficients (*P*-value < 0.05), and arrows with black numbers indicate nonsignificant pathway coefficients. The pathways coefficient from baseline MRS to follow-up IL-6 is significant (*P*-value = 0.027)

We assessed the temporal relationship between BMI-associated methylation with inflammation markers (interleukin 6, IL-6 and Tumor necrosis factor alpha, TNF-alpha) and 155 metabolites using CLPM to determine if the BMI-associated methylation  may influence inflammation or metabolism. Within the 116 BMI-associated CpGs identified, nine CpGs showed significant path coefficient (*P*-value < 0.05) from baseline methylation to follow-up IL-6, while six CpGs showed significant path coefficient (*P*-value < 0.05) from baseline IL-6 to follow-up methylation. No CpGs showed significant path coefficient with *P*_FDR_ < 0.05 significance. We further generated a methylation risk score (MRS) which reflected the combined effect of the 116 identified BMI, and examined the causal relationship between MRS and IL-6 (Fig. 3). The path coefficient from baseline MRS to follow-up IL-6 was significant (*P*-value = 0.023). There was no significant causal direction indicated in the analysis of TNF-alpha.

In the analyses of the 155 metabolomic biomarkers, no significant pathway direction was suggested by the CLPM under *P*_FDR_ < 0.05. At a significant level of *P*-value < 0.05, we found four lipoproteins with causal pathways from baseline MRS to follow-up metabolites: large high-density lipoproteins (HDL) carrying total lipids, large HDL carrying free cholesterol, extreme small very-low-density lipoprotein (VLDL) carrying cholesteryl esters, and intermediate-density lipoproteins (ILD) carrying cholesteryl esters (Additional file 1: Table S9).

## Discussion

Using EWAS analysis in 409 multi-ethnic Asian participants, we identified 116 BMI-associated CpGs and 8 WC-associated CpGs at epigenome-wide significance, of which six BMI-associated CpGs and two WC-associated CpGs are novel. We replicated some of the well-established CpGs associated with BMI consistently across different Asian populations. Seven out of eight identified WC-associated CpGs were also associated with BMI at *P*_FDR_ < 0.05, and two CpGs cg15103625 in *RSRC1* and cg07421368 in *ETAA1* were found novel in both WC and BMI associations, indicating common methylation sites influenced by both general and abdominal obesity. Out of the 116 BMI-associated CpGs reported in this study, we also identified six novel CpGs while 110 have been previously reported in other populations. Over 90% of the significant CpGs reported by Wahl et al. [24] and Sun et al. [25] showed consistency in direction of effect, indicating shared CpG associations across populations. We also observed moderate consistency when comparing our findings in other populations. This may indicate possible unique signals in Asian population, or by chance due to the limited samples size.

To determine if the changes in methylation might influence gene expression in blood, we performed cis-expression association analysis on the 116 identified CpGs. However, no CpGs were found to be significantly associated with gene expression after Bonferroni correction. Although some BMI-associated CpGs identified in Europeans were reported to influence gene expression in blood [22, 24], such association may not be detectable given our modest sample size. Another possible reason could be that we used microarray to profile expression, which could only profile predefined genes through hybridization.

Some of the CpGs identified in this study were in loci known to be involved in adiposity. cg02871985 located in the CpG island near *THADA* was negatively associated with BMI in our EWAS (*P*-value = 4.75 × 10^–6^). Although the association might be influenced by a common SNP rs33979934 at 109 bp away from the CpG, the SNP-CpG association was not significant as reported by a current mQTL study in iOmic samples [32]. *THADA* is a regulator of energy consumption and energy storage and has been associated with cold adaption [33]. Numerous SNPs in *THADA* have been reported to be associated with adiposity and T2D in multi-ethnic GWAS studies [34–36]. Studies in *Drosophila* have shown that *THADA* triggers thermogenesis by uncoupling ATP hydrolysis from calcium transport into the endoplasmic reticulum [33]. Our study suggested the methylation level at *THADA* might be downstream of BMI, highlighting the effect of methylation in the mechanism of obesity and T2D. However, lack of expression association results limits our ability to make a definitive causal inference, and more studies are needed to explore the biological mechanism of methylation in *THADA*. In addition to the novel associations, we also replicated previously reported BMI-associated CpGs. For example, cg05511958 in *CHCHD5* shows significant association with BMI (*P*-value = 4.29 × 10^–7^) in our results. Recently, *CHCHD5* has been reported to be associated with hypertension and obesity in a Chinese population [37].

In our EWAS, there are also some CpGs identified in loci not previously linked to adiposity. Three of the novel adiposity-associated CpGs cg15103625 in *RSRC1*, cg07421368 in *ETAA1*, and cg08010984 in *TNIK* are in genes involved in serine metabolism. *RSRC1* encodes arginine- and serine-rich proteins that plays an important role in multiple cellular functions by altering RNA splicing, while *ETAA1* and *TNIK* are involved in protein serine/threonine kinase activator activity. *TNIK* encoded a serine/threonine kinase that functions as an activator of the Wnt signaling pathway [38]. In our study, cg15103625 in *RSRC1* and cg07421368 in *ETAA1* were significantly associated with both BMI and WC. GWAS in Europeans identified adiposity-associated genetic variants in all three loci [39–41]. However, the association between adiposity and serine metabolism remains unclear.

Of these six novel CpGs, cg15103625 and cg08010984 were not presented in the 450 k array, which could explain why they were not identified in previous studies. The Illumina Infinium HumanMethylation EPIC array provides more coverage as compared to the 450 k array [42], thus allowing for the identification of potentially novel BMI-associated CpG sites. Due to lack of expression association evidence, we do not have sufficient evidence to confirm the effect of the identified adiposity-associated CpGs on the nearby candidate genes, and we would suggest cautious interpretation of our results.

DNA methylation can be jointly influenced by genetic and environmental exposures. One of the most well-established modifiable factors that influence DNA methylation is cigarette smoking. Many EWAS have identified and replicated smoking-associated CpGs across ethnicities [43, 44], where most of the identified signals showed hypomethylation in smokers than nonsmokers. In our study, all MEC Chinese samples were nonsmokers whereas smoking prevalence ranged from 22 to 39% in the iOmics participants. To reduce the confounding by differences in smoking status, we included smoking as one of the covariates in the regression model in all analyses of iOmics samples, including EWAS, expression association analysis, and MR.

Obesity as a chronic disease is also affected by environmental factors such as diet and physical activity. Thus, the causal relationship between DNA methylation and BMI has been an area of research interest in recent years [22, 24, 25]. In this study we examined the temporal relationship between BMI and methylation using CLPM and bidirectional MR. By applying CLPM in 124 Chinese individuals, we found 92 CpGs with significant path coefficients from baseline BMI to follow-up DNA methylation compared to none in the reverse direction. This result suggests that BMI may be the cause, rather than consequence of DNA methylation. While our MR analyses did not identify CpGs supporting causality in either direction, possibly due to the small sample size, the correlation between predicted and observed BMI-CpG association was stronger in the backward MR, indicating a higher possibility of BMI being the causal factor. The evidence from our CLPM is consistent with previous studies using MR across populations [22, 24].

Chronic low-grade inflammation and an activation of the immune system are commonly reported to be associated with the pathogenesis of obesity-related insulin resistance and T2D [45, 46]. The inflammatory disturbances in the obese adipocyte are reflected by elevated pro-inflammatory cytokines such as IL-6 [47] and TNF-alpha [48, 49]. Our longitudinal analysis supports the association between methylation and IL-6 by providing inferred causation from obesity-associated methylation to IL-6. Our findings help to strengthen the hypothesis that methylation may be involved in the mechanism of obesity-related inflammatory perturbation; however, further causal inference analyses will be needed to validate the evidence.

Our study is an Asian-focused EWAS study that included both cross-sectional and longitudinal analyses. The primary analysis was performed using data from cross-sectional design, and the causal relationship was analyzed using the longitudinal design. There are also some notable limitations to our study. First, we recognize that our sample size is limited for EWAS. However, we found good consistency in our results compared with previous EWAS studies. Second, DNA methylation was measured using DNA extracted from whole blood rather than adipose tissue. DNA methylation in blood samples can directly capture the methylation changes in immune system such as memory lymphocytes or leukocytes, which are involved in inflammation or immune response pathways [50]. Moreover, EWAS of age has shown highly concordant associations in different tissues and blood samples [51], and that multiple BMI-associated CpGs have been replicated in both leukocyte and adipose tissues [26].

## Conclusion

Our study identified and replicated common adiposity-associated CpGs reported in other populations. We also identified six novel CpGs associated with BMI and two with WC that reached epigenome-wide significance in Asian populations. We further reported evidence of the causal effect of BMI on the identified methylation sites using a longitudinal setting in a Chinese population. In addition, the causal analyses also indicate that BMI-associated methylation might play a role in inflammatory and lipoprotein-related biological pathways. Our findings help to prioritize relevant methylation site for future functional studies and provide a foundation for further research to study the mechanism of obesity-related diseases through the influence of BMI on DNA methylation.

## Methods

### Study population

All samples included in this study were selected from the MEC [30]. The first set of samples were from iOmics which included 286 MEC participants (102 Chinese, 86 Malay, 98 Indian) at their first follow-up that were randomly selected through age-stratified and gender-stratified sampling [31]. All samples have epigenome-wide methylation measured on the Illumina Infinium HumanMethylation EPIC array. A subset of 258 participants (95 Chinese, 79 Malay, 84 Indian) has genome-wide genotype data (Illumina 2.5 M microarray genotyping) and 208 (78 Chinese, 53 Malay, 77 Indian) samples have gene expression data (Affymetrix Human Gene 1.0 ST arrays). The second set of samples were 140 Chinese randomly selected healthy participants in MEC with epigenome-wide methylation profiled on the same EPIC array at both their baseline and first follow-up, hereafter referred to as ‘MEC Chinese sample’ set. Mean follow-up time for all participants was 6.8 years (SD = 1.39). Samples at first follow-up were used in epigenome-wide association analyses of obesity measures, whereas baseline samples were only used in causal relationship analysis (Additional file 1: Table S1). All participants completed a detailed interview, physical examination, and provided blood samples at each visit. Anthropometric measures such as height, weight, and waist and hip circumference were measured during the physical examination. Age, gender, smoking and alcohol drinking patterns, medication history, and other covariates were collected through detailed interviews. An overview of the samples and omics measurements is provided in Fig. 1. All participants provided written formed consent, and all protocols associated with the study were approved by the National University of Singapore Institutional Review Board.

### DNA methylation quantification and quality control

DNA methylation was quantified in bisulfite-converted genomic DNA using Illumina Infinium HumanMethylation EPIC array from buffy coats in two separate laboratories for the iOmics and MEC Chinese samples. Quality control was performed separately for each set of samples using the same protocol. Raw signal intensities were retrieved using R package *minfi*. To remove background noise from the dyes, we performed background correction using *bgcorrect.illumina* function in *minfi*. For each probe, detection *P*-value was computed as the probability of the total signal (methylation + unmethylated) being detected above the background signal level, as estimated from negative-control probes. Small detection *P*-values indicate higher probability of true signal compared to background noise. Samples with high probe missingness defined as detection *P*-value > 1 × 10^–16^ in > 5% in all probes were excluded (MEC Chinese, *n* = 13; iOmics, *n* = 0). We computed the median intensity of probes on chromosomes X and Y separately and derived gender information from the probes where difference of log2 median intensity > − 2 indicates male, and difference of < − 2 indicates female. Samples with discordant gender information compared to self-reported gender were excluded (MEC Chinese, *n* = 3; iOmics, *n* = 5). We then performed quality control at probe level for the remaining samples. Probes with detection *P*-value > 1 x 10^–16^ in > 5% of the samples (MEC Chinese, *n* = 42,987; iOmics, *n* = 12,503) or beadcounts < 3 in > 5% samples (MEC Chinese, *n* = 1,884; iOmics, *n* = 85) were excluded. Probes mapping to sex chromosomes were also excluded (MEC Chinese, *n* = 17,237; iOmics, *n* = 18,710). We also flagged cross-reactive probes (MEC Chinese, *n* = 40,242; iOmics, *n* = 41,468) and probes with genetic marker information (MEC Chinese, *n* = 26,682; iOmics, *n* = 28,641). Quantile normalization was performed on the probes separately for the MEC Chinese baseline and follow-up samples, and by ethnicity in iOmics samples to adjust for technical variability. After quality control, there were 281 iOmics samples (100 Chinese, 84 Malays, 97 Indians) with 834,881 probes and 264 MEC Chinese (136 at baseline and 128 at follow-up) with 821,032 probes for subsequent analyses (Additional file 1: Table S2). Finally, the methylation level at each probe site was represented as a beta ($\beta$) value ranging from 0 (nonmethylated) to 1 (completely methylated). The beta value was defined as the ratio of the methylated signal (M) to the sum of methylated and unmethylated signal (U), $\beta$=M/(M + U + 100).

To account for batch effect and technical variability, we performed principal component (PC) analysis on 635 control probes that are internal probes in Illumina BeadChips that cover steps such as bisulfite conversion, normalization, and hybridization. The PCs derived from the control probes were included as covariates in subsequent regression models. Blood cell composition were also estimated based on the Houseman algorithm [52] separately for both sets of samples. The proportion of granulocytes, monocytes, B cells, CD4+ T cells, CD8+ T cells, and natural killer cells were subsequently included as covariates in regression models to reduce cell-type confounding.

### Metabolomics biomarkers quantification

Metabolomics biomarkers were quantified in the 140 MEC Chinese samples at baseline and follow-up using targeted metabolomics approaches in a high-throughput proton nuclear magnetic resonance metabolomics platform (Nightingale Health, Helsinki, Finland) [53]. A total of 155 biomarkers were measured, and they can be broadly classified into cholesterol markers (*n* = 9), glycerides and phospholipids (*n* = 9), apolipoproteins (*n* = 3), fatty acids (*n* = 16), amino acids (*n* = 8), lipoproteins (*n* = 101), glycolysis-related metabolites (*n* = 3), ketone bodies (*n* = 3), and markers of fluid balance (*n* = 2) and inflammation (*n* = 1). Values below detection limit were replaced by 0.9 times the minimum of the remaining values.

### Inflammation markers measurements

Two inflammatory markers, IL-6 and TNF-alpha, were measured in 140 MEC Chinese plasma samples at baseline and follow-up. IL-6 levels were measured using the Quantikine HS Human IL-6 Immunoassay (R&D Systems, Cat NO: HS600C). TNF-alpha levels was measured using TNF-alpha Ultrasensitive enzyme‐linked immunosorbent assay (ALPCO, Cat No: 45-TNFHUU-E01). The immunoassays were performed according to the manufacturer’s instructions, and duplicate tests were done for multiple samples to ensure consistency of the results. Values below or beyond detection limit were imputed using fitted value from the linear regression plot of the optical density and concentration in each plate.

### Association between adiposity and DNA methylation

Epigenome-wide associations of BMI and WC with DNA methylation were performed separately by ethnicity within the iOmics and MEC Chinese samples. Linear regression models were used with DNA methylation as independent variable, and BMI/WC as dependent variables. Untransformed BMI/WC and beta value were used in the regression model for ease of interpretation. To adjust for confounders, age, sex, smoking habit, blood cell type composition, and first five control probe PCs (sensitivity analysis indicated that the first five control probe PCs explained a large proportion of the variation; Additional file 2: Figure S1) were included as covariates in the model. Smoking habit was defined as a binary variable (0 = never smoke, 1 = ever smoke).

The association results from each sample set and ethnicity were meta-analyzed using inverse-variance fixed-effects meta-analysis implemented via *meta* package in R. CpGs with *P*_FDR_ < 0.05 were considered significantly associated with BMI/WC. For comparison between significant BMI-associated CpGs in our meta-analysis and previously reported CpGs, we queried MRC-IEU EWAS Catalog [54] and Atlas Catalog [55] for all BMI and WC associated CpGs identified in previous studies. We define novel CpGs as CpGs that are more than 500 kb away from known CpG-BMI or CpG-WC associations from either catalogs. Obesity-related genes were defined as genes that were reported in GWAS of BMI in NHGRI-EBI GWAS Catalog [56] or Public Health Genomics and Precision Health Knowledge Base [57].

To compare previously reported EWAS results in European data with our multi-ethnic Asian data, we performed reciprocal lookups of (1) previously reported BMI and WC-associated CpGs in our meta-analysis, namely 10,261 European/South Asians in Wahl et al. (*n* = 254 CpGs) [24] and 1965 Europeans/African Americans in Sun et al. (*n* = 349 CpGs) [25]; (2) BMI-associated and WC-associated CpGs identified in our meta-analysis in additional samples from KORA from the first follow-up of the S3 survey (European: KORA F3, *n* = 484), the first and second follow-ups of the S4 survey (European: KORA F4, *n* = 1,709 on 450 k array; European: KORA FF4, *n* = 1,874 on EPIC array, respectively) and LOLIPOP (South Asian: LOLIPOP, *n* = 2,680 on 450 k array) [24]. We evaluated the consistency in direction of association between the studies and calculated the proportion of CpGs with the same direction of effect using binomial test of null hypothesis that the proportion is equal to 0.5.

### Association between DNA methylation and gene expression

Expression data were available for a subset of iOmics samples (*n* = 208: 78 Chinese, 53 Malay, 77 Indian) [31]. RNA was collected from whole blood samples in each ethnicity, with gene expression quantified using the Affymetrix Human Gene 1.0 ST arrays (Affymetrix Inc., Santa Clara, CA). Gene expression quality control and normalization were performed in each ethnicity separately. These included (1) exclusion of lowly expressed genes, defined as genes with expression levels less than the mean expression level of control probes in more than 90% of the samples; (2) variance stabilization [58] and quantile normalization to standardize the distribution of expression levels across samples; (3) accounting for known and unknown experimental confounders by adjusting for sex, batch effects and five probabilistic estimation of expression residuals (PEER) factors [59, 60]; and (4) a rank inverse normal transformation of the residuals. Approximately 15,000 autosomal gene expression probes remained in each ethnicity (*n* = 15,268 in Chinese, *n* = 15,187 in Malay, *n* = 15,302 in Indian) and were used in subsequent analyses. We performed association test of BMI-associated CpGs with transcripts located within 1 Mb of the corresponding CpGs in 208 iOmics samples, adjusting for age, blood cell composition, and control probe PCs. The results were analyzed separately by ethnicity, adjusting for age, blood cell composition, and control probe PCs. The results were combined using fixed effect inverse-variance meta-analysis.

### Temporal relationship analysis of BMI and DNA methylation

To explore the temporal relationship of BMI and DNA methylation, we performed (1) CLPM on the MEC Chinese with methylation data at two timepoints (*n* = 124 each at baseline and follow-up), and (2) bidirectional two-sample MR in a subset of 208 iOmic samples with genotype available (78 Chinese, 53 Malay, 77 Indian).

#### Cross-lagged panel model (CLPM)

In CLPM, both baseline and follow-up BMI were adjusted for age and sex by regression residual analysis and Z-standardized. DNA methylation was also adjusted and standardized with further adjustment for blood cell composition and five control PCs. Pearson correlation and regression coefficients were estimated from the models, and validity of model fitting was evaluated by the comparative fit index [61]. Structural equations used in CLPM are: (1) Baseline BMI ~ Baseline DNA methylation; (2) Follow-up DNA methylation ~~ Baseline DNA methylation + Baseline BMI + e1; (3) Follow-up DNA ~~ Baseline DNA methylation + Baseline BMI + e2, where ~ indicates correlation and ~~ indicates regression; e1 and e2 are error terms. All CLPM analyses were performed using Lavaan package in R.

#### Bidirectional Mendelian randomization (MR)

We selected cis-SNPs associated with 116 BMI-associated CpGs as instrumental variable. We first selected SNPs within 1 Mb of the 116 BMI-associated CpGs (*n* = 211,727) and performed linear regression analysis using CpGs as dependent variable and SNPs as independent variable, adjusting for covariates used in the discovery analysis. The analysis was performed for each ethnicity and combined using fixed-effect meta-analysis. The top associated SNP (i.e., SNP with the smallest P-value) for each CpG was chosen as IV (*n* = 116). SNPs with nonsignificant association (*P*-value > 0.05, *n* = 1), SNPs within probe-binding sequence (*n* = 2), and SNPs associated with BMI after adjusting for CpGs (*n* = 8) were excluded. Out of the remaining 105 SNPs, the effect size of 94 SNPs on BMI could be obtained in the GWAS summary statistics in over 170,000 Japanese [16]. The predicted effect ($eff_{CpG {\text{-}} BMI}^{2}$) and standard error ($SE_{CpG {\text{-}} BMI}$) of CpG on BMI was calculated as below [24]:

$$\begin{aligned} eff_{CpG {\text{-}} BMI}^{2} & = \frac{{eff_{BMI {\text{-}} SNP}^{2} }}{{eff_{CpG {\text{-}} SNP}^{2} }} \\ SE_{CpG {\text{-}} BMI} & = \sqrt {\frac{{eff_{BMI {\text{-}} SNP}^{2} }}{{eff_{CpG {\text{-}} SNP}^{2} }}*\left( {\frac{{SE_{BMI -{\text{-}}SNP}^{2} }}{{eff_{BMI {\text{-}} SNP}^{2} }} + \frac{{SE_{CpG {\text{-}} SNP}^{2} }}{{eff_{CpG {\text{-}} SNP}^{2} }}} \right)} \\ \end{aligned}$$

The predicted effect was then compared with the observed effect from the EWAS results using correlation test. To assess reverse causality, we used PRS as IV. PRS was calculated as the weighted sum of effect of 85 BMI-associated SNPs identified in Japanese [16], using score function in Plink V1.9 [62]. We used a Bonferroni-adjusted *P*-value threshold of 5 × 10^–4^ to account for multiple testing.

### Association between DNA methylation with inflammation markers and metabolomics biomarkers

To examine the association and causality between BMI-associated methylation with inflammation markers and metabolomics biomarkers, we calculated a methylation risk score (MRS) to reflect the combined effect of the 116 identified BMI-associated CpGs. CLPM was used to study the temporal relationship between the BMI-associated MRS and two inflammation (IL-6 and TNF-alpha) and 155 metabolomics biomarkers. Linear regression model was used to study the association between MRS and inflammation and metabolomics biomarkers. Inflammation and metabolomics biomarkers with significant associations with the MRS were selected for CLPM to study the causality. Inflammation and metabolomics biomarkers were regressed on age, sex, and batch. MRS was regressed on age, sex, blood cell composition, and five control PCs. Both inflammation and metabolomics biomarkers and the MRS were Z-standardized. The CLPM was further adjusted for follow-up time.

## Supplementary Information

**Additional file 1:** **Table S1-S9**. **Table S1**. Summary clinical characteristics of samples. **Table S2**. Quality control (QC) of DNA methylation data. **Table S3**. Epigenome-wide association results of obesity-associated CpGs (P_FDR_<0.05) in Asian samples by sample set, ethnicity and meta-analyses. **Table S4**. Summary of lookups of previously reported association results in our trans-ethnic meta-analysis. **Table S5**. Summary of lookups of association results from our trans-ethnic meta-analysis in KORA and LOLIPOP. **Table S6**. Pathway coefficients in cross-lagged panel model (CLPM) with 116 BMI-associated CpGs for BMI and methylation. **Table S7**.  Results of forward Mendelian randomization for methylation as the cause. **Table S8**. Results of backward Mendelian randomization for BMI as the cause. **Table S9**. Pathway coefficients in cross-lagged panel model (CLPM) with 116 BMI-associated CpGs for metabolomics biomarkers and methylation risk score.

**Additional file 2: Figure S1**. Sensitivity analysis of control probes principal components analysis. The first five principal components were included in regression models.

## Data Availability

The datasets used and/or analyzed during the current study are available from the corresponding author on reasonable request.

## Abbreviations

BMI: Body mass index
CLPM: Cross-lagged panel model
EWAS: Epigenome-wide association study
FDR: False discovery rate
GWAS: Genome-wide association study
HDL: High-density lipoproteins
IDL: Intermediate-density lipoproteins
IL-6: Interleukin 6
iOmics: The Singapore Integrative Omics Study
IV: Instrumental variable
KORA: Cooperative Health Research in the Region of Augsburg
LOLIPOP: The London Life Sciences Prospective Population Study
MEC: Multi-Ethnic Cohort
MRS: Methylation risk score
PC: Principal component
PEER: Probabilistic estimation of expression residuals
PRS: Polygenic risk score
QC: Quality control
TNF-alpha: Tumor necrosis factor alpha
T2D: Type2 diabetes
VLDL: Very-low-density lipoprotein
WC: Waist circumference

## References

[1] Ramachandran A, Snehalatha C. Rising burden of obesity in Asia. J Obes. 2010;2010. 10.1155/2010/868573 PMC2939400 20871654

[2] Jaacks LM, Vandevijvere S, Pan A, McGowan CJ, Wallace C, Imamura F (2019). The obesity transition: stages of the global epidemic. Lancet Diabetes Endocrinol.

[3] Polsky S, Ellis SL (2015). Obesity, insulin resistance, and type 1 diabetes mellitus. Curr Opin Endocrinol Diabetes Obes.

[4] Riobo SP (2013). Obesity and diabetes. Nutr Hosp.

[5] Seravalle G, Grassi G (2017). Obesity and hypertension. Pharmacol Res.

[6] Lavie CJ, Milani RV, Ventura HO (2009). Obesity and cardiovascular disease: risk factor, paradox, and impact of weight loss. J Am Coll Cardiol.

[7] Nazare JA, Smith JD, Borel AL, Haffner SM, Balkau B, Ross R (2012). Ethnic influences on the relations between abdominal subcutaneous and visceral adiposity, liver fat, and cardiometabolic risk profile: the International Study of Prediction of Intra-Abdominal Adiposity and Its Relationship With Cardiometabolic Risk/Intra-Abdominal Adiposity. Am J Clin Nutr.

[8] Vazquez G, Duval S, Jacobs DR, Silventoinen K (2007). Comparison of body mass index, waist circumference, and waist/hip ratio in predicting incident diabetes: a meta-analysis. Epidemiol Rev.

[9] Huxley R, James WP, Barzi F, Patel JV, Lear SA, Suriyawongpaisal P (2008). Ethnic comparisons of the cross-sectional relationships between measures of body size with diabetes and hypertension. Obes Rev.

[10] Sluik D, Boeing H, Montonen J, Pischon T, Kaaks R, Teucher B (2011). Associations between general and abdominal adiposity and mortality in individuals with diabetes mellitus. Am J Epidemiol.

[11] Hill JO, Peters JC (1998). Environmental contributions to the obesity epidemic. Science.

[12] Scuteri A, Sanna S, Chen WM, Uda M, Albai G, Strait J, et al. Genome-wide association scan shows genetic variants in the FTO gene are associated with obesity-related traits. PLoS Genet. 2007;3(7):e115. 10.1371/journal.pgen.0030115 PMC1934391 17658951

[13] Frayling TM, Timpson NJ, Weedon MN, Zeggini E, Freathy RM, Lindgren CM (2007). A common variant in the FTO gene is associated with body mass index and predisposes to childhood and adult obesity. Science.

[14] Organization WH. Obesity and overweight 2020 [Available from: https://www.who.int/news-room/fact-sheets/detail/obesity-and-overweight.

[15] Locke AE, Kahali B, Berndt SI, Justice AE, Pers TH, Day FR (2015). Genetic studies of body mass index yield new insights for obesity biology. Nature.

[16] Akiyama M, Okada Y, Kanai M, Takahashi A, Momozawa Y, Ikeda M (2017). Genome-wide association study identifies 112 new loci for body mass index in the Japanese population. Nat Genet.

[17] Yengo L, Sidorenko J, Kemper KE, Zheng Z, Wood AR, Weedon MN (2018). Meta-analysis of genome-wide association studies for height and body mass index in approximately 700000 individuals of European ancestry. Hum Mol Genet.

[18] Jones PA (2012). Functions of DNA methylation: islands, start sites, gene bodies and beyond. Nat Rev Genet.

[19] Dragic D, Ennour-Idrissi K, Michaud A, Chang SL, Durocher F, Diorio C (2020). Association Between BMI and DNA Methylation in Blood or Normal Adult Breast Tissue: A Systematic Review. Anticancer Res.

[20] Aslibekyan S, Demerath EW, Mendelson M, Zhi D, Guan W, Liang L (2015). Epigenome-wide study identifies novel methylation loci associated with body mass index and waist circumference. Obesity (Silver Spring).

[21] Al Muftah WA, Al-Shafai M, Zaghlool SB, Visconti A, Tsai PC, Kumar P (2016). Epigenetic associations of type 2 diabetes and BMI in an Arab population. Clin Epigenetics.

[22] Mendelson MM, Marioni RE, Joehanes R, Liu C, Hedman AK, Aslibekyan S, et al. Association of Body Mass Index with DNA Methylation and Gene Expression in Blood Cells and Relations to Cardiometabolic Disease: A Mendelian Randomization Approach. PLoS Med. 2017;14(1):e1002215. 10.1371/journal.pmed.1002215 PMC5240936 28095459

[23] Martin DI, Cropley JE, Suter CM (2011). Epigenetics in disease: leader or follower?. Epigenetics.

[24] Wahl S, Drong A, Lehne B, Loh M, Scott WR, Kunze S (2017). Epigenome-wide association study of body mass index, and the adverse outcomes of adiposity. Nature.

[25] Sun D, Zhang T, Su S, Hao G, Chen T, Li QZ (2019). Body Mass Index Drives Changes in DNA Methylation: A Longitudinal Study. Circ Res.

[26] Demerath EW, Guan W, Grove ML, Aslibekyan S, Mendelson M, Zhou YH (2015). Epigenome-wide association study (EWAS) of BMI, BMI change and waist circumference in African American adults identifies multiple replicated loci. Hum Mol Genet.

[27] Guay SP, Brisson D, Lamarche B, Marceau P, Vohl MC, Gaudet D (2013). DNA methylation variations at CETP and LPL gene promoter loci: new molecular biomarkers associated with blood lipid profile variability. Atherosclerosis.

[28] Na YK, Hong HS, Lee WK, Kim YH, Kim DS (2015). Increased methylation of interleukin 6 gene is associated with obesity in Korean women. Mol Cells.

[29] Balakrishnan A, Guruprasad KP, Satyamoorthy K, Joshi MB (2018). Interleukin-6 determines protein stabilization of DNA methyltransferases and alters DNA promoter methylation of genes associated with insulin signaling and angiogenesis. Lab Invest.

[30] Tan KHX, Tan LWL, Sim X, Tai ES, Lee JJ, Chia KS, et al. Cohort Profile: The Singapore Multi-Ethnic Cohort (MEC) study. Int J Epidemiol. 2018;47(3):699-j. 10.1093/ije/dyy014 29452397

[31] Saw WY, Tantoso E, Begum H, Zhou L, Zou R, He C (2017). Establishing multiple omics baselines for three Southeast Asian populations in the Singapore Integrative Omics Study. Nat Commun.

[32] Kassam I, Tan S, Gan FF, Saw WY, Tan LW, Moong DKN (2021). Genome-wide identification of cis DNA methylation quantitative trait loci in three Southeast Asian Populations. Hum Mol Genet.

[33] Moraru A, Cakan-Akdogan G, Strassburger K, Males M, Mueller S, Jabs M, et al. THADA Regulates the Organismal Balance between Energy Storage and Heat Production. Dev Cell. 2017;41(1):72–81 e6. 10.1016/j.devcel.2017.03.016 PMC5392496 28399403

[34] Zhao W, Rasheed A, Tikkanen E, Lee JJ, Butterworth AS, Howson JMM (2017). Identification of new susceptibility loci for type 2 diabetes and shared etiological pathways with coronary heart disease. Nat Genet.

[35] Vujkovic M, Keaton JM, Lynch JA, Miller DR, Zhou J, Tcheandjieu C, et al. Discovery of 318 new risk loci for type 2 diabetes and related vascular outcomes among 1.4 million participants in a multi-ancestry meta-analysis. Nat Genet. 2020;52(7):680–91. 10.1038/s41588-020-0637-y PMC7343592 32541925

[36] Lotta LA, Wittemans LBL, Zuber V, Stewart ID, Sharp SJ, Luan J (2018). Association of Genetic Variants Related to Gluteofemoral vs Abdominal Fat Distribution With Type 2 Diabetes, Coronary Disease, and Cardiovascular Risk Factors. JAMA.

[37] Wu L, Gao L, Zhao X, Zhang M, Wu J, Mi J. A new risk locus in CHCHD5 for hypertension and obesity in a Chinese child population: a cohort study. BMJ Open. 2017;7(9):e016241. 10.1136/bmjopen-2017-016241 PMC5722095 28893745

[38] Masuda M, Uno Y, Ohbayashi N, Ohata H, Mimata A, Kukimoto-Niino M (2016). TNIK inhibition abrogates colorectal cancer stemness. Nat Commun.

[39] Pulit SL, Stoneman C, Morris AP, Wood AR, Glastonbury CA, Tyrrell J (2019). Meta-analysis of genome-wide association studies for body fat distribution in 694 649 individuals of European ancestry. Hum Mol Genet.

[40] Kichaev G, Bhatia G, Loh PR, Gazal S, Burch K, Freund MK (2019). Leveraging Polygenic Functional Enrichment to Improve GWAS Power. Am J Hum Genet.

[41] Zhu Z, Guo Y, Shi H, Liu CL, Panganiban RA, Chung W (2020). Shared genetic and experimental links between obesity-related traits and asthma subtypes in UK Biobank. J Allergy Clin Immunol.

[42] Pidsley R, Zotenko E, Peters TJ, Lawrence MG, Risbridger GP, Molloy P (2016). Critical evaluation of the Illumina MethylationEPIC BeadChip microarray for whole-genome DNA methylation profiling. Genome Biol.

[43] Christiansen C, Castillo-Fernandez JE, Domingo-Relloso A, Zhao W, El-Sayed Moustafa JS, Tsai PC (2021). Novel DNA methylation signatures of tobacco smoking with trans-ethnic effects. Clin Epigenetics.

[44] Joehanes R, Just AC, Marioni RE, Pilling LC, Reynolds LM, Mandaviya PR (2016). Epigenetic Signatures of Cigarette Smoking. Circ Cardiovasc Genet.

[45] Zeyda M, Stulnig TM (2009). Obesity, inflammation, and insulin resistance–a mini-review. Gerontology.

[46] Esser N, Legrand-Poels S, Piette J, Scheen AJ, Paquot N (2014). Inflammation as a link between obesity, metabolic syndrome and type 2 diabetes. Diabetes Res Clin Pract.

[47] Eder K, Baffy N, Falus A, Fulop AK (2009). The major inflammatory mediator interleukin-6 and obesity. Inflamm Res.

[48] Hotamisligil GS, Spiegelman BM (1994). Tumor necrosis factor alpha: a key component of the obesity-diabetes link. Diabetes.

[49] Tzanavari T, Giannogonas P, Karalis KP (2010). TNF-alpha and obesity. Curr Dir Autoimmun.

[50] Bauer M (2018). Cell-type-specific disturbance of DNA methylation pattern: a chance to get more benefit from and to minimize cohorts for epigenome-wide association studies. Int J Epidemiol.

[51] Horvath S (2013). DNA methylation age of human tissues and cell types. Genome Biol.

[52] Houseman EA, Accomando WP, Koestler DC, Christensen BC, Marsit CJ, Nelson HH (2012). DNA methylation arrays as surrogate measures of cell mixture distribution. BMC Bioinformatics.

[53] Soininen P, Kangas AJ, Wurtz P, Suna T, Ala-Korpela M (2015). Quantitative serum nuclear magnetic resonance metabolomics in cardiovascular epidemiology and genetics. Circ Cardiovasc Genet.

[54] Bristol Uo. MRC-IEU EWAS Catalog 2020 [Available from: http://www.ewascatalog.org.

[55] Li M, Zou D, Li Z, Gao R, Sang J, Zhang Y (2019). EWAS Atlas: a curated knowledgebase of epigenome-wide association studies. Nucleic Acids Res.

[56] Buniello A, MacArthur JAL, Cerezo M, Harris LW, Hayhurst J, Malangone C (2019). The NHGRI-EBI GWAS Catalog of published genome-wide association studies, targeted arrays and summary statistics 2019. Nucleic Acids Res.

[57] Yu W, Gwinn M, Dotson WD, Green RF, Clyne M, Wulf A (2016). A knowledge base for tracking the impact of genomics on population health. Genet Med.

[58] Huber W, von Heydebreck A, Sultmann H, Poustka A, Vingron M (2002). Variance stabilization applied to microarray data calibration and to the quantification of differential expression. Bioinformatics.

[59] Parts L, Stegle O, Winn J, Durbin R. Joint genetic analysis of gene expression data with inferred cellular phenotypes. PLoS Genet. 2011;7(1):e1001276. 10.1371/journal.pgen.1001276 PMC3024309 21283789

[60] Stegle O, Parts L, Durbin R, Winn J. A Bayesian framework to account for complex non-genetic factors in gene expression levels greatly increases power in eQTL studies. PLoS Comput Biol. 2010;6(5):e1000770. 10.1371/journal.pcbi.1000770 PMC2865505 20463871

[61] Joreskog KG (1996). Modeling development: using covariance structure models in longitudinal research. Eur Child Adolesc Psychiatry.

[62] Chang CC, Chow CC, Tellier LC, Vattikuti S, Purcell SM, Lee JJ (2015). Second-generation PLINK: rising to the challenge of larger and richer datasets. Gigascience.

