# Supplementary figures and images for: Impact of BMI and waist circumference on epigenome-wide DNA methylation and identification of epigenetic biomarkers in blood: an EWAS in multi-ethnic Asian individuals
Source: Clin Epigenetics. 2021 Oct 20;13:195. doi: 10.1186/s13148-021-01162-x (PMC8527674; doi:10.1186/s13148-021-01162-x)

Variances

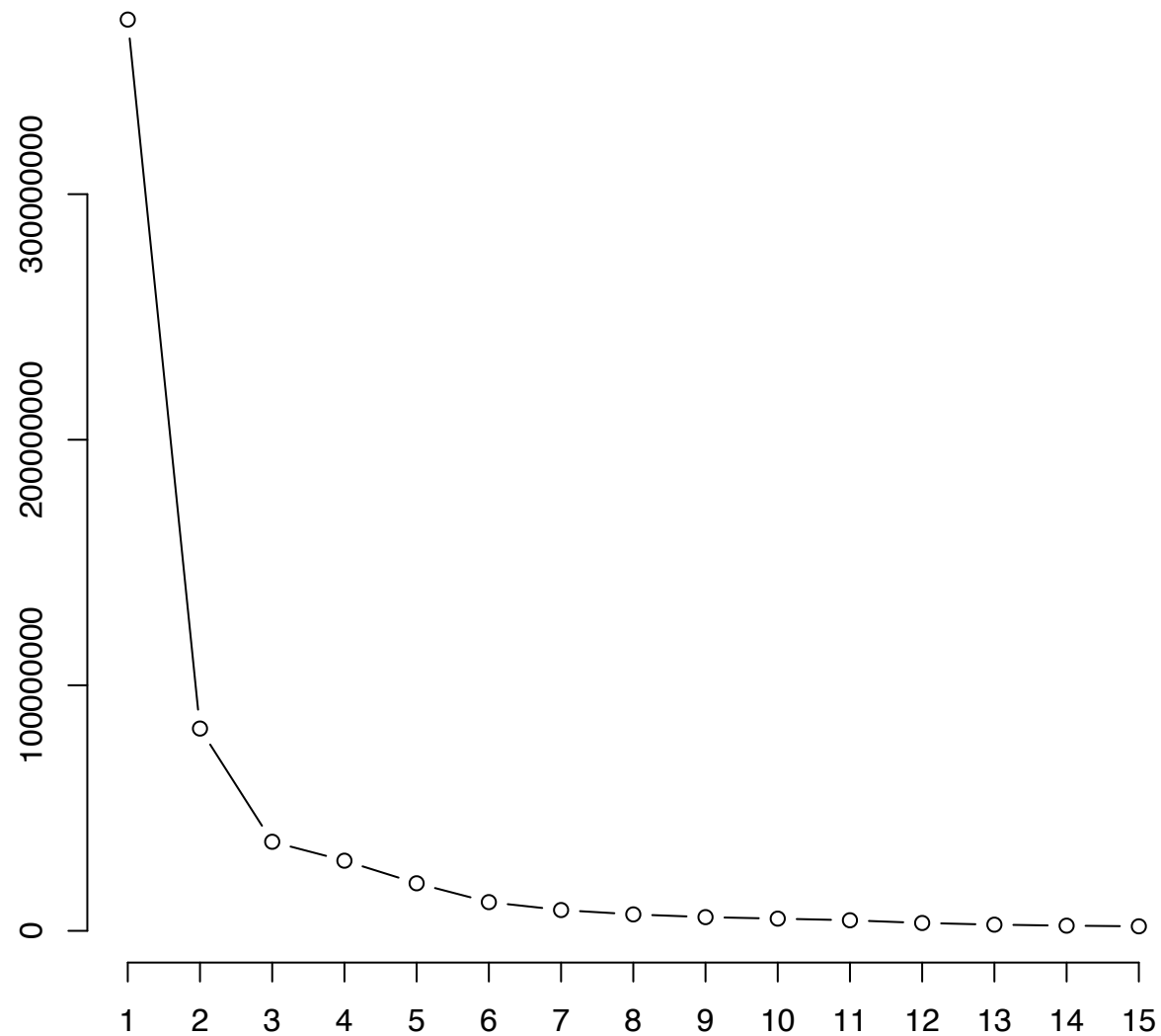

MEC Chinese Control Probe PCs

Variances

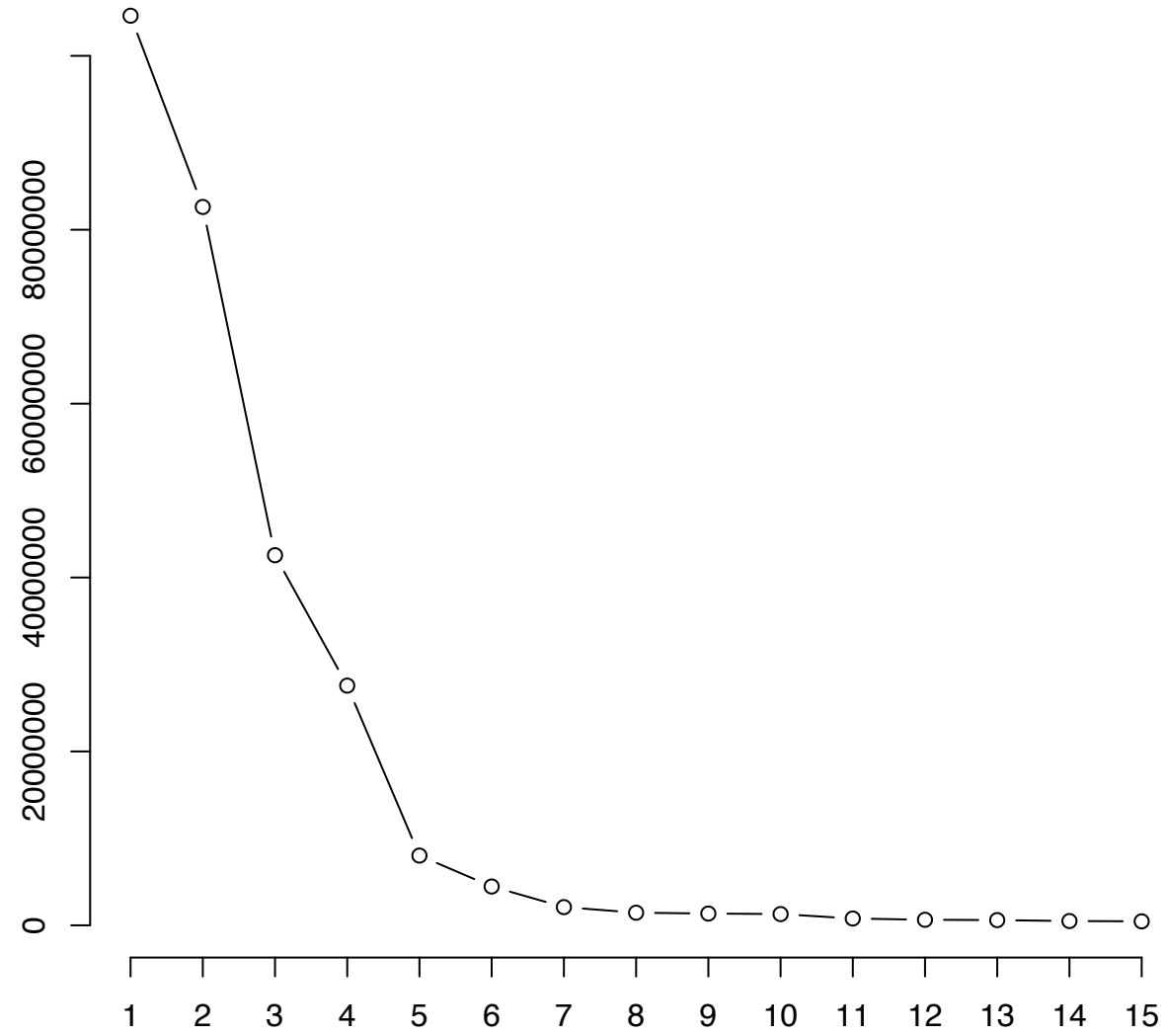

iOmics Control Probe PCs

Supplement: Supplementary file 2 — Additional file 2: Figure S1. Sensitivity analysis of control probes principal components analysis. The first five principal components were included in regression models. [file 13148_2021_1162_MOESM2_ESM.pdf]
